# Targets and Potential Mechanism of *Scutellaria baicalensis* in Treatment of Primary Hepatocellular Carcinoma Based on Bioinformatics Analysis

**DOI:** 10.1155/2022/8762717

**Published:** 2022-02-12

**Authors:** Defu Liu, Zhengjun Wang, Li Zhong, Caoyu Xie, Xiaonan Huang, Yaofeng Zhi, Yuzhuo Zhang, Jiaying Liang, Zhenni Shi, Jin Huang, Shuhe Zhang, Jin Zhang, Fuping Ding

**Affiliations:** ^1^Guangzhou University of Chinese Medicine, School of Basic Medical Sciences, Guangzhou, China; ^2^Guangzhou University of Chinese Medicine, Research Center of Integrative Medicine, School of Basic Medical Sciences, Guangzhou, China; ^3^Shenzhen Hospital, Guangzhou University of Chinese Medicine (Futian), Guangzhou, China; ^4^Guangdong New South Stem Cell Regenerative Medicine Technology Co., Ltd, Guangzhou, China; ^5^Guangzhou University of Chinese Medicine, School of Nursing, Guangzhou, China

## Abstract

**Objective:**

To analyze the target and potential mechanism of *Scutellaria baicalensis* (SB) in the treatment of HCC based on bioinformatics, so as to provide suggestions for the diagnosis, treatment, and drug development of hepatocellular carcinoma (HCC).

**Methods:**

The regulated gene targets of SB were screened by gene expression pattern clustering and differential analysis of gene expression data of HepG2 cells treated with SB at 0 h, 1 h, 3 h, 6 h, 12 h, and 24 h. The module genes related to HCC were identified by the weighted gene coexpression network analysis (WGCNA). KEGG and GO enrichment were used to analyze the molecular function and structure of the module, and GSEA was used to evaluate the different functional pathways between normal people and patients with HCC. Then, the module gene was used for univariate Cox proportional hazard analysis and the least absolute shrinkage and selection operator (LASSO) Cox regression analysis to build a prognostic model. The protein-protein interaction network (PPI) was used to analyze the core genes regulated by SB (CGRSB) of the module, and the survival curve revealed the CGRSB impact on patient survival. The CIBERSORT algorithm combined with correlation analysis to explore the relationship between CGRSB and immune infiltration. Finally, the single-cell sequencing technique was used to analyze the distribution of CGRSB at the cellular level.

**Results:**

SB could regulate 903 genes, of which 234 were related to the occurrence of HCC. The prognosis model constructed by these genes has a good effect in evaluating the survival of patients. KEGG and GO enrichment analysis showed that the regulation of SB on HCC mainly focused on some cell proliferation, apoptosis, and immune-related functions. GSEA enrichment analysis showed that these functions are related to the occurrence of HCC. A total of 24 CGRSB were obtained after screening, of which 13 were significantly related to survival, and most of them were unfavorable factors for patient survival. The correlation analysis of gene expression showed that most of CGRSB was significantly correlated with T cells, macrophages, and other functions. The results of single-cell analysis showed that the distribution of CGRSB in macrophages was the most.

**Conclusion:**

SB has high credibility in the treatment of HCC, such as CDK2, AURKB, RRM2, CENPE, ESR1, and PRIM2. These targets can be used as potential biomarkers for clinical diagnosis. The research also shows that the p53 signal pathway, MAPK signal pathway, apoptosis pathway, T cell receptor pathway, and macrophage-mediated tumor immunity play the most important role in the mechanism of SB in treating HCC.

## 1. Introduction

Liver cancer is a malignant disease with high incidence in the population, of which hepatocellular carcinoma (HCC) takes up about 80% for the most common [1]. According to a statistical report provided by the International Agency for Research on Cancer of the World Health Organization in 2020, liver cancer is the sixth most common cancer in the world, with a mortality rate of the top three of all tumors, and a 5-year survival rate of less than 10%. It is one of the tumors with the worst prognosis of all cancers [2, 3]. Although many drugs have been approved for clinical use, due to the lack of accurate targets in the current treatment of liver cancer, serious drug side effects, and the gradual drug resistance produced by tumor cells in the course of treatment, the therapeutic effect has been greatly reduced. It is difficult to improve the survival of patients [4].

Chinese medicine is a multicomponent therapy based on holism. As a complementary alternative therapy for all kinds of cancer, Chinese medicine has a broad-spectrum antitumor effect of regulating immunity and promoting apoptosis [5]. Compared with other treatments, Chinese medicine has the advantages of low cost and less adverse reactions in the treatment of liver cancer. At the same time, the efficacy and safety of Chinese medicine in the treatment of liver cancer has been confirmed in many clinical studies [6, 7]. Studies have shown that Chinese medicine can enhance tumor immunity, alleviate the primary factors of early HCC, and improve the survival rate and quality of life in patients with advanced HCC [8, 9]. *Scutellaria baicalensis* (SB) is a kind of Chinese herb, which belongs to the genus Scutellaria of Labiatae with bitter taste and cold nature. It has the effects of clearing heat and protecting liver, purging fire and detoxification, stopping blood, and calming fetus that has been widely used as a medicine in China for thousands of years [10]. With the in-depth study of SB, it has been found that baicalin, baicalein, wogonoside, and wogonin play an important role in antitumor, which is mainly related to apoptosis and proliferation. However, its specific mechanism has not been thoroughly found [11]. Clinical studies have shown that baicalin combined with percutaneous transcatheter arterial chemoembolization (TACE) can significantly reduce the adverse reactions and prolong the survival time of patients with liver cancer compared to the control group [12]. Therefore, further reveal of the mechanism in SB treating liver cancer is necessary for clinical drug development and use.

Due to the complexity of the role of traditional Chinese medicine, the traditional research methods are difficult to reveal its mechanism. Bioinformatics is a new discipline that uses mathematical tools to mine useful information from massive biological data. Based on bioinformatics, this study discussed the mechanism of SB in the treatment of patients with liver cancer from gene to protein level, which provides a theoretical basis for follow-up research and development and experimental verification.

## 2. Materials and Methods

### 2.1. Data Source and Processing

The HCC transcriptional data were sequenced from the TCGA database [13] (https://portal.gdc.cancer.gov/), including 50 normal cases and 371 HCC cases. The clinical data were downloaded and extracted at the same time. The gene expression data (GSE84783) of HepG2 hepatoma cells treated with SB at 0 h, 1 h, 3 h, 6 h, 12 h, and 24 h were obtained from the GEO database (https://www.ncbi.nlm.nih.gov/geo/) [14]. The limma package [15] in R4.0.5 is used to standardize the original data. The single-cell sequencing dataset (GSE146115) of patients with HCC was also from the GEO database. The data were filtered by Seurat package in R4.0.5 to screen the cells with more than 200 genes, and the genes expressed in more than 3 cells. The mitochondrial and ribosomal gene contents were calculated, so that the mitochondrial gene content was less than 5 and the ribosomal gene content was less than 50. Then, normalization and dimensionality reduction of PCA, tSNE, and UMAP were carried out, and the batch correction of CCA was judged according to the results of dimensionality reduction [16]. HCC expression data from the GEO database were used for external verification (GSE76427).

### 2.2. Discovery of Potential Target Genes of SB

By using the Mfuzz package [17] in R4.0.5, the sequencing data of SB treating HepG2 were clustered and visualized according to gene expression patterns at different times. The samples treated with 0 h, 1 h, and 3 h of SB were divided into the short-term treatment group and 6 h, 12 h, and 24 h, the others. The differences were analyzed by limma package and screened according to *P* < 0.05 and |logFC| > 0.5. Then, the differential genes were intersected with the clustering results, and the intersected genes will be the targeted genes of SB.

### 2.3. Modules of WGCNA Related to Hepatocarcinogenesis in SB

Using the WGCNA package [18] in R4.0.5, the weighted gene coexpression network (WGCNA) was constructed from the expression matrix of intersected genes, and the Pearson correlation coefficient between genes was calculated to determine the best soft threshold to approximate the scale-free network. After that, the matrix was transformed into a topological overlap matrix (TOM), and the degree of dissimilarity between genes was calculated for hierarchical clustering. Finally, the dynamic shearing method is used to merge similar modules, and the modules with high correlation and significant differences are selected.

### 2.4. Enrichment Analysis of HCC Genes Targeted by SB

The gene ontology database (GO, http://geneontology.org/) [19] and KEGG database (https://www.kegg.jp/kegg/pathway.html) [20] were used for enrichment of genes, setting *P* < 0.05, |logFC| > 0.5 as standard, using clusterProfiler package in R4.0.5. Three modules were contained in GO analysis: cell component (CC), molecular function (MF), and biological process (BP). KEGG was for pathway enrichment. GSEA4.0.1 software was used to for gene sets enrichment analysis, setting “c2.cp.kegg.v7.4.symbols.gmt” and “h.all.kegg.v7.4.symbols.gmt” as reference gene sets, with the normal group and HCC group as the standard of classification. Enrichment analysis was carried out according to default parameter setting (no collapse), and the number of random combinations was 1000.

### 2.5. Construction and Evaluation of the Prognostic Model

Survival package [21] in R4.0.5 was used for univariate cox proportional hazard analysis of key genes, filtering out genes significantly associated with prognosis, calculating hazard ratio (HR), and drawing forest map. Subsequently, Lasso regression analysis was performed to determine final genes related to prognosis and survival [22]. The train sets were used to construct the risk score model related to the prognosis of HCC based on the linear combination of gene expression level and regression coefficient. Among them, the calculation formula of patient risk score is as follows:

(1) $$\begin{array}{c} \text{Risk score}=\sum \text{coefficents}\times \text{expression levels}. \end{array}$$

The median risk score was used as threshold to divide patients into the high-risk group and low-risk group, drawing Kaplan–Meier survival curve [23]. It was worth emphasizing that the random grouping process was executed by createDataPartition function. The receiver operating characteristic (ROC) curves of age [24], gender, stage and grade, and risk score in the training set were plotted by survival ROC package, and the area under the ROC curve (AUC) [25] was calculated to evaluate prediction accuracy of the model. The test set performed the same analysis for internal validation queue to verify. AUC >0.7 indicated that the constructed model had good prediction performance.

### 2.6. Core Targets Revealed by Protein-Protein Interaction in the Regulatory Network of SB

Uploaded genes in WGCNA significant modules to STRING 11.0 [26] database for constructing the protein-protein interaction (PPI) network and remove unconnected nodes. CytoNCA [27] plug-in in Cytoscape 3.8.0 was used to analyze topological properties. According to betweenness centrality (BC), degree centrality (DC), and closeness centrality (CC), the CGRSBs were screened. Molecular docking was used to explore the interaction between CGRSB and baicalin, baicalein, wogonoside, and wogonin, the four main components of SB. Preparation for docking is as follows: get receptor proteins for docking using the AlphaFold database (https://alphafold.ebi.ac.uk/) [28] and remove nonstandard amino acids, hydrogenate, and charge. Ligand molecules for docking were obtained through PubChem database (https://pubchem.ncbi.nlm.nih.gov/) [29], and energy minimization was executed. AutoDock Vina software is used for the final molecular docking [30].

### 2.7. Survival Analysis of CGRSBs

Combining CGRSBs gene expression data and clinical information, using limma, survival, and survminer package in R4.0.5, the clinical samples were grouped according to median gene expression, and survival curve was drawn in batches. *P* < 0.05 was considered statistically significant.

### 2.8. Immune Infiltration Revealed the Correlation and Coexpression between Core Genes and Immune Cells

The CIBERSORT deconvolution algorithm [31] in R4.0.5 was used to calculate relative proportion of 22 tumor infiltrating immune cells. According to the filtering results of *P* < 0.05, the ggplot2 package in R4.0.5 was used for visualization. Then, the median immune cell level was used as threshold to divide data into the high infiltration group and low infiltration group. Survival and survminer packages were used for survival analysis to evaluate the correlation between the infiltration mode of immune cells in HCC and prognosis. To be same, *P* < 0.05 was considered statistically significant.

### 2.9. Genome-Wide Single-Cell Sequencing Analysis Revealed Distribution of CGRSB in Cell

The first 11 principal components (PC) were selected for visualization, and principal component analysis (PCA) was used to reduce the dimension of feature space. The K-nearest neighbor (KNN) classification algorithm was used to determine the best resolution according to FindCluster function to cluster the cells. The unified manifold approximation and projection (UMAP) method was used to visualize the clustering results after dimension reduction. The singleR package was used to preliminarily annotate the clustering results, and the annotation results were manually corrected in combination with the literature. Finally, the distribution of CGRSBs in each cell group was visualized (details in S1).

## 3. Results

### 3.1. Potential Regulatory Targets of SB

**Figure 1:**
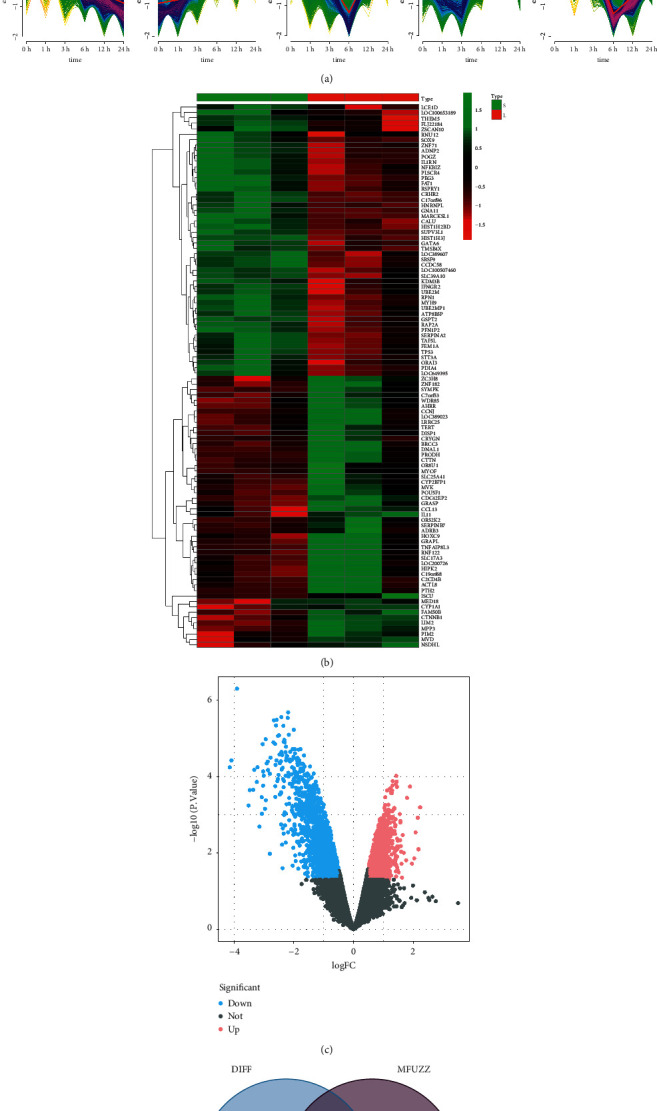
(a) Cluster diagram of expression pattern, a total of 10 clusters, abscissa for different time, ordinate for gene expression. (b) Heatmap of the top 100 differentially expressed genes in drug process data in the normal group and HCC group. (c) Volcano map of difference analysis in drug process data; red represents upregulated genes, blue represents downregulated genes, and grey represents no significant difference genes. (d) Venn diagram of intersection between differential genes and gene expression patterns clusters 6 and 7.

Under the process of SB, genes with different expression patterns were clustered into 10 groups, in which the expression patterns of 6 and 7 gene sets had obvious patterns with the expression of the former decreased, while the expression of the latter was contrary along the prolongation of drug treatment processing (Figure 1(a)). Therefore, the two gene sets were selected for further analysis. A total of 3210 differentially expressed genes were screened by differential analysis between the early and late stages of drug treatment (Figures 1(b) and 1(c)), and a total of 903 drug-adjustable targets were obtained after intersection with clustering 6 and 7 (Figure 1(d)).

### 3.2. SB Regulated HCC-Related Targets

**Figure 2:**
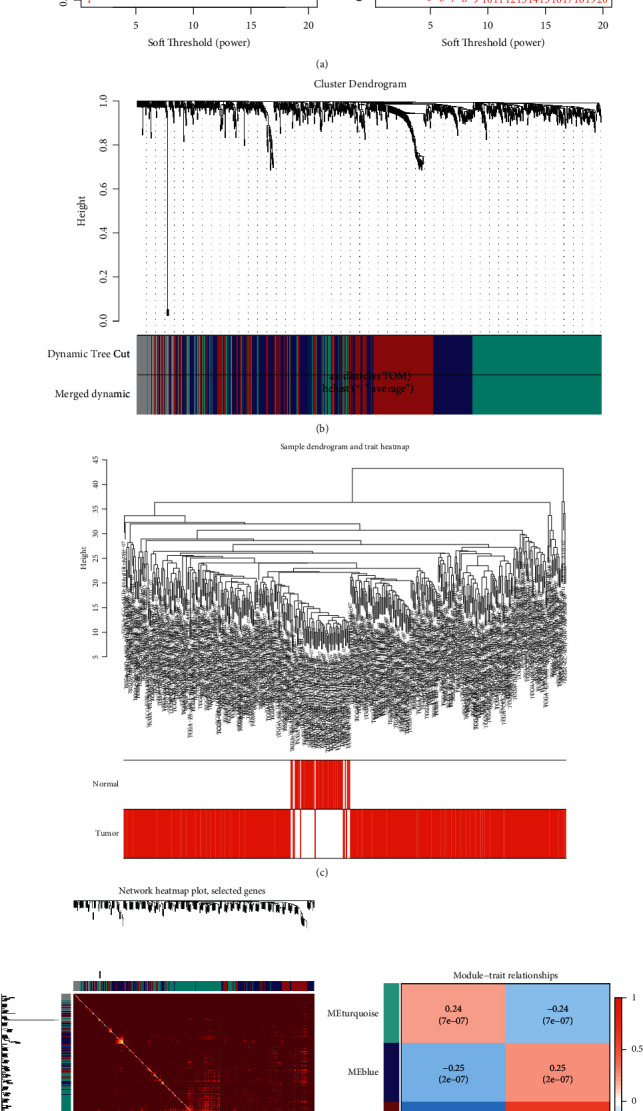
(a) The horizontal axis of the two graphs is soft threshold, the vertical axis of the left graph is the square of the correlation coefficient in the network, and the vertical axis of the right graph represents the mean value of all gene adjacency function in the module. (b) Gene clustering tree constructed by the one-step method and modules obtained before and after cutting. (c) Heatmap of sample clustering. (d) Heatmap of module vector gene TOM matrix. (e) Heatmap of correlation between modules and phenotypes.

WGCNA applied soft threshold of 4 to construct a scale-free network through function confirmation, so the *β* value was greater than 0.9 and connectivity was the minimum value in the platform period (Figure 2(a)). In the sample clustering heatmap, there was high differentiation between the normal group and HCC group, indicating that there was a certain heterogeneity between the two groups (Figure 2(c)). Genes were clustered into four modules. It could be seen that the diagonal color of TOM matrix heatmap was deeper, indicating that the topology overlap of genes in the module was high and the clustering results were relatively clear (Figure 2(d)). Finally, the MEbrown module of the four modules had the highest correlation with tumor (0.61), and the *P* value (2e-44) was the most significant (Figure 2(e)). Therefore, the genes in MEbrown module were identified as HCC-related targets regulated by drugs and used for subsequent analysis.

### 3.3. Evaluation in Function of SB Regulating HCC

**Figure 3:**
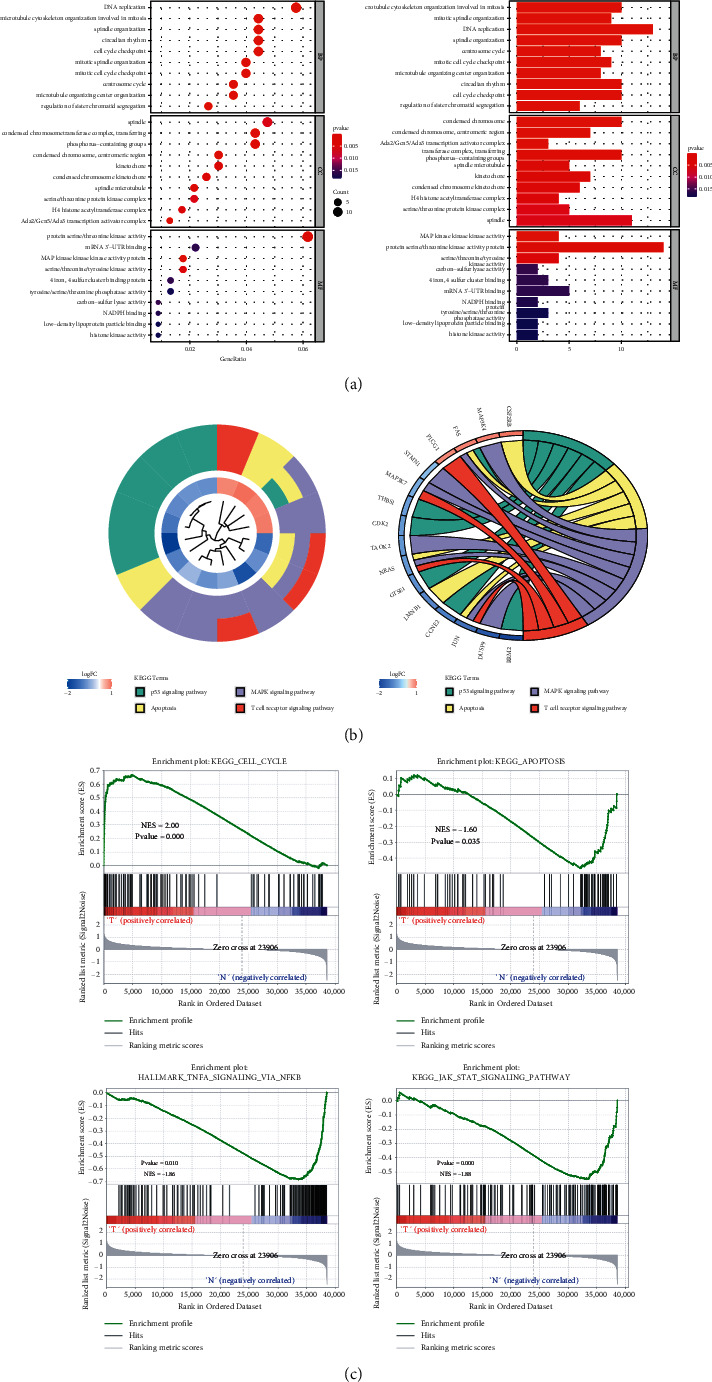
(a) GO enrichment map; dot size in left and column length in right represents the number of enriched genes; red and blue represent high and low significance. (b) The left graph is the clustering tree of KEGG-enriched genes and pathways, and the right graph is the circle map of four pathways in KEGG enrichment. (c) GSEA enrichment analysis results of four pathways.

GO enrichment analysis of MEbrown module showed that at the BP level, the genes in the MEbrown module were mainly involved in the processes related to cell cycle, mitosis, and DNA replication; at the CC level, they were mostly related to spindle and chromosome structure; while at the MF level, they were mainly related to kinase activity, such as serine/threonine kinase activity and adenosine phosphokinase activity (Figure 3(a)). In the results of KEGG enrichment analysis, the p53 signal pathway, apoptosis pathway, T cell receptor pathway, and MAPK signal pathway were significantly enriched by 16 genes (Figure 3(b)). They appeared simultaneously in the results of GSEA analysis, which was statistically significant (Figure 3(c)).

### 3.4. Construction of the Prognostic Model

**Figure 4:**
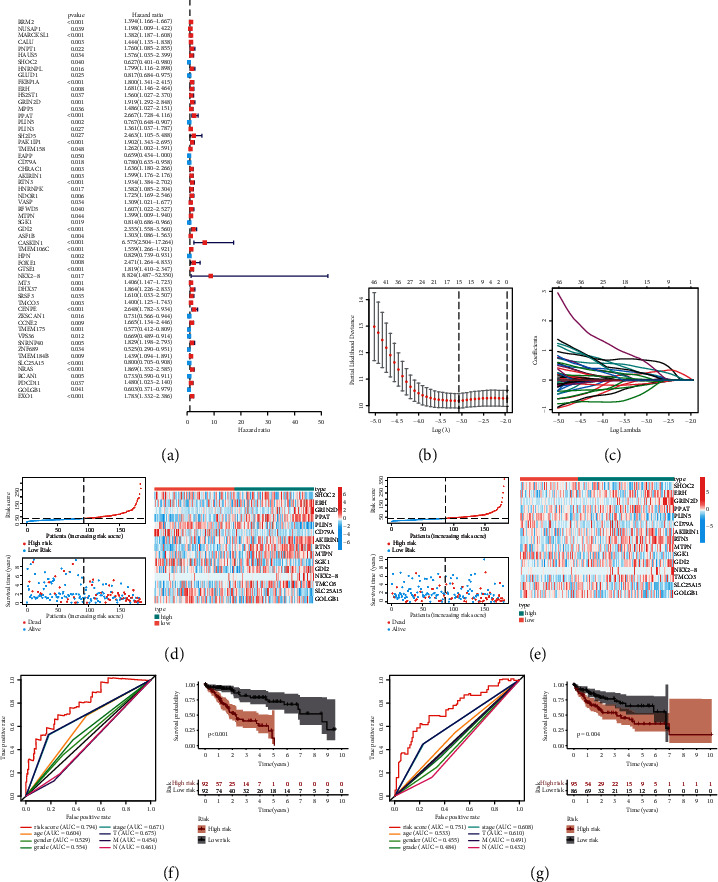
(a) Forest map of univariate cox proportional hazard analysis. (b) The variation of mean square error (MSE) with parameter ln*λ*. (c) Coefficients changes with parameters of genes involved in the prognostic model of Lasso regression, 0 means deletion. (d) Risk curve, survival, and gene expression of different risk patients in the train group. (e) Risk curve, survival, and gene expression of different risk patients in the test group. (f) ROC map and survival curve of the train group. (g) ROC map and survival curve of the test group.

**Figure 5:**
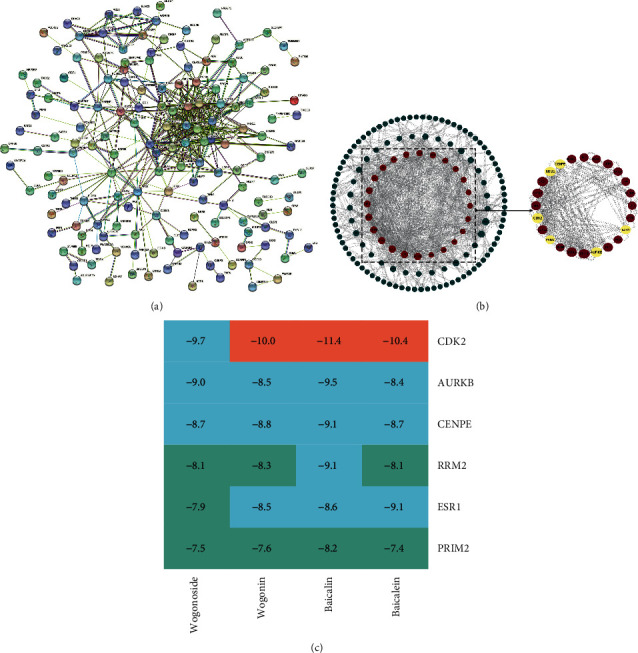
(a) PPI network in genes of MEbrown module. (b) Active components of SB-HCC mutual target network diagram, the line represents the existence of interaction, red represents the CGRSBs, and yellow represents the higher score node in the CGRSBs. (c) Results for molecular docking.

A total of 57 genes significantly related to the prognosis were screened by univariate COX regression analysis, and most of them were adverse factors affecting the prognosis, which mean the HR was greater than 1 (Figure 4(a)). The Lasso regression model was constructed based on 15 of these genes (Figures 4(b) and 4(c)), which showed that there was a significant difference in patient survival among the high and low-risk groups assessed by the model (Figures 4(f) and 4(g)). In addition, the AUC under the ROC curve of the training group and the internal test group was 0.794 and 0.751, respectively, which were significantly larger than the area under the ROC curve of other clinical traits, which indicated that the performance of the prognosis model was good (Figures 5(a) and 5(b)). The external dataset validates our results (S2).

### 3.5. Determination of Core Targets in SB Regulation Networks

The results of PPI showed that HCC-related genes regulated by SB had a certain network, and *P* value of network enrichment was far less than 0.05, with 421 edges in 234 nodes (Figure 5(a)). After screening the PPI network, 24 CGRSBs were obtained, which were CDK2, MYBL2, ESR1, AURKB, JUN, RRM2, KIF11, ASF1B, LMNB1, CCNE2, CENPE, EXO1, PRIM2, CDK9, DUT, CALM1, KAT2A, NRAS, RUVBL1, HNRNPL, ERH, SAFB, PLCG1, DDX55, and more importance existed in CDK2, AURKB, RRM2, CENPE, ESR1, and PRIM2 (Figure 5(b)). Molecular docking showed that they had high binding possibility, all binding energy less than −7. Among them, CDK2 has the most stable combination with baicalin, baicalein, wogonoside, and wogonin (Figure 5(c)).

### 3.6. Survival Evaluation and Difference Validation of CGRSBs

**Figure 6:**
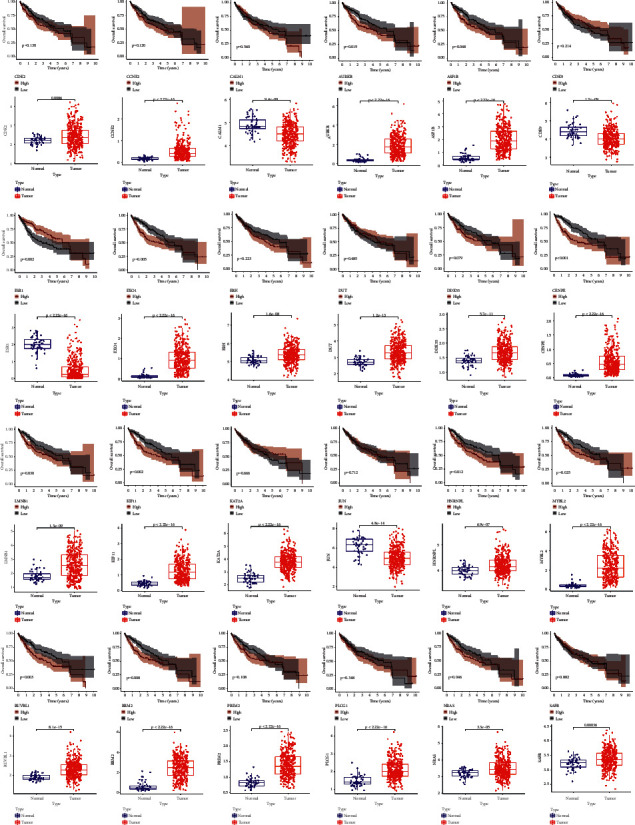
Survival curve and differential box diagram of each gene in CGRSBs.

The expression of 24 CGRSBs were significantly different between normal and HCC patients (Figure 6). Not only that, they also had a certain influence on survival, among which 13 are statistically significant, namely, ASF1B, AURKB, CENPE, ESR1, EXO1, HNRNPL, KIF11, LMNB1, MYBL2, NRAS, RRM2, and RUVBL1. In addition, the expression of most genes had a negative effect on patient survival, with the survival rate of patients in the low expression group being higher than that of patients in the high expression group. The external dataset had similar results (S2).

### 3.7. Immune Infiltration Level of Patients and Its Relationship with CGRSBs

**Figure 7:**
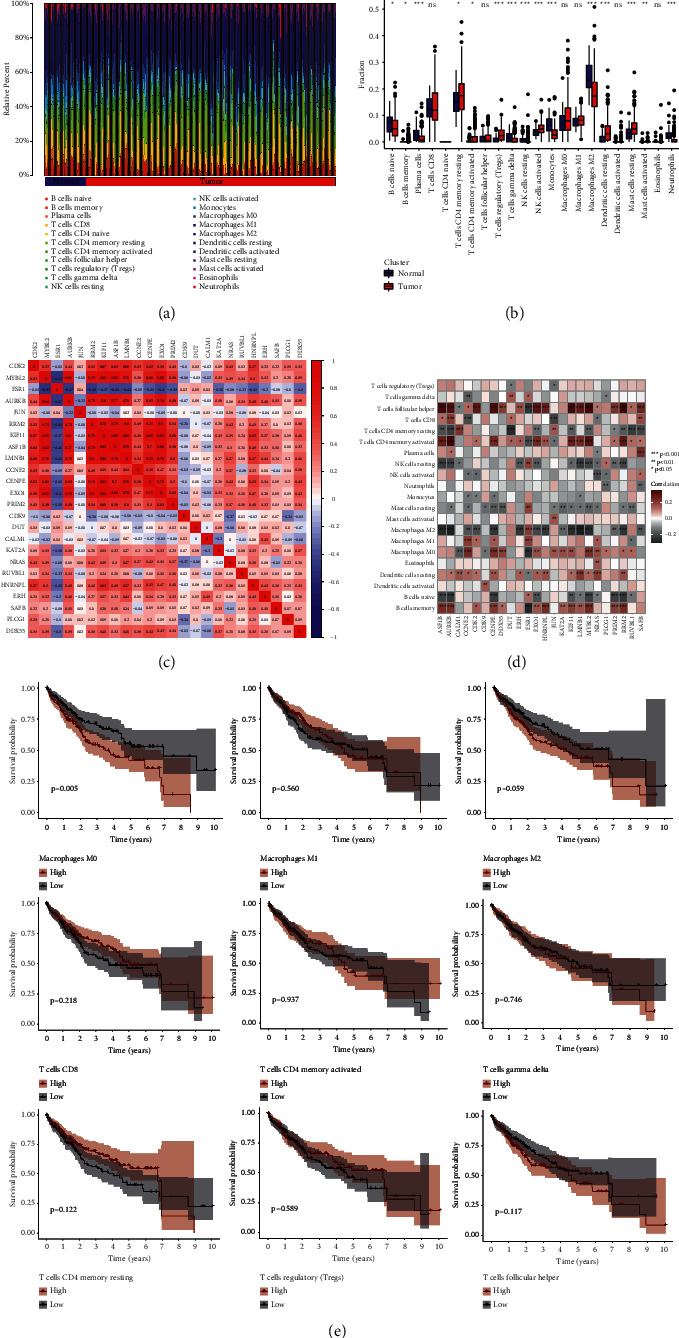
(a) Infiltration of different immune cells in the HCC group and normal group. (b) Analysis of the difference of immune cell activity between the HCC group and normal group. (c) Correlation diagram of coexpression of core genes; blue represents negative correlation and red represents positive correlation. (d) Heatmap of correlation between CGRSB and immune cells. (e) Survival curves of different immunocytes.

The results of immune infiltration showed that naive B cells, plasma cells, T cell CD4 memory resting, T cell CD4 memory activation, T cell regulation, T cell *γδ*, natural killer (NK) cell activation, NK cell resting, monocytes, macrophages M2, dendritic cell resting, mast cell resting, mast cell activation, neutrophil infiltration in HCC, and normal tissues were statistically significant (Figures 7(a) and 7(b)). The correlation analysis of CGRSBs showed that CGRSBs had the same expression pattern and were basically positively correlated (Figure 7(c)). Studies showed that the functions of T cells and macrophages were significantly associated with CGRSB (Figure 7(d)), suggesting that SB could regulate the function of immune cells by regulating the expression of core genes, which was consistent with our previous enrichment results. These results were validated in external datasets (S2). In addition, survival analysis showed that the proportion of infiltration of these cells was correlated with survival (Figure 7(e)).

### 3.8. Expression Patterns of Core Targets in SB at Cellular Level

**Figure 8:**
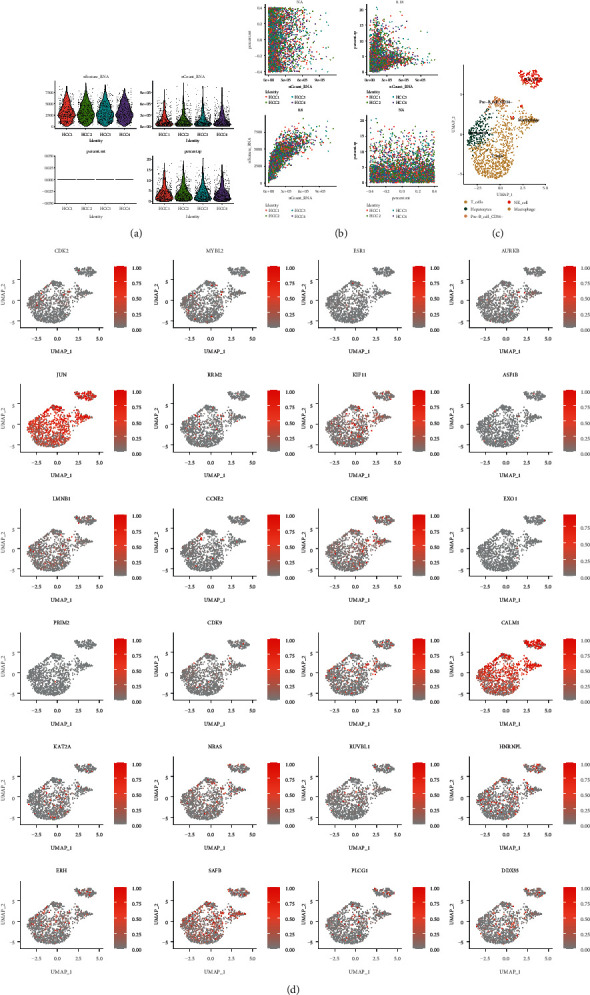
(a) Quality control diagram of single-cell sequencing data from different patients. (b) Correlation scatter plot of total sequencing, mitochondrial, and ribosomal genes in different patients. (c) Cell cluster annotation diagram. (d) The distribution of CGRSB in different cell clusters.

A total of 1263 cells were obtained after quality control, and the first 11 PCs were selected for dimension reduction according to standard deviation and *P* value. Then, 697 T cells, 230 hepatocytes, 157 NK cells, 81 macrophages, and 68 B cells (Figure 8(c)) were annotated. There were some differences in the distribution of CGRSBs in various types of cells, and the CGRSBs concentrated in macrophages are the most; they are CENPE (*P*=1.05*E* − 08, avg_log2FC = 0.34), NRAS (*P*=0.0002, avg_log2FC = 0.33), HNRBPL (*P*=0.0082, avg_log2FC = 0.25), and SAFB (*P*=0.0005, avg_log2FC = 0.44), 4 in total (Figure 8(d)).

## 4. Discussion

**Figure 9:**
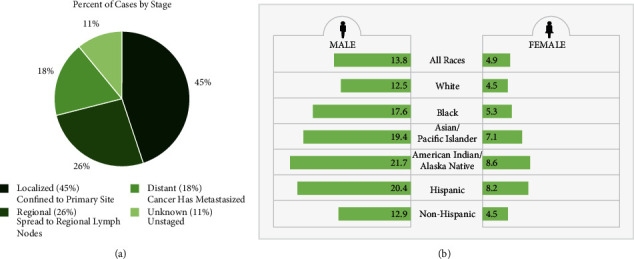
Picture captured from the SEER database.

According to the statistics in the Surveillance, Epidemiology, and End Results (SEER) database [32] of the National Cancer Institute (NCI), HCC patients are mainly diagnosed in the early stage, and the proportion of male patients of different races is higher than that of women (Figures 9(a) and 9(b)). The data downloaded from TCGA in this study had the same characteristics, with a total of 175 patients in stage 1 (49.6%) and more male patients than female patients (255 males and 122 females). This shows that the data obtained in this study are representative, and the results are reliable.

In order to better confirm the relationship between SB and genes in the network, we clustered and extracted genes with the same trend in time variables, observed the changes of each clustering result in the overall time dimension to determine which gene groups have a certain upward and downward trend before and after drug application, and speculated that these may be drug-targeted regulatory genes. On the other hand, the difference analysis can screen out the up and downregulated genes with statistical significance more accurately, which reduces the possibility of false positive results. WGCNA analysis selected the MEbrown modules related to the occurrence of liver cancer and constructed the prognosis model. In the comparison of the effectiveness of the model, the model constructed by the MEbrown module gene has a stronger ability to predict the prognosis than other clinical traits and can be used to evaluate the prognosis of clinical patients.

Previous studies had shown that SB can delay the progression of HCC by regulating cell cycle, promoting apoptosis and autophagy, and inhibiting cancer cell metastasis and drug resistance [33–38]. KEGG and GO enrichment results show that downstream proteins of genes regulated by SB can participate in the p53 signaling pathway, apoptosis pathway, T cell receptor pathway, MAPK signaling pathway, and cell cycle-related processes. This is consistent with the previous research results, which furtherly confirm that SB had an important effect on cell proliferation and apoptosis.

Studies show that tumor suppressor protein p53 is a key participant in tumor suppression, which can promote cell growth arrest, apoptosis and senescence, and block angiogenesis [39–41]. p53 can also promote antitumor microenvironment to inhibit tumor occurrence.By releasing related factors, the hepatic stellate cell expressing p53 made macrophage polarization tend to M1 state, which can inhibit the occurrence of tumor, while the factors secreted by p53 deficient stellate cells stimulated macrophage polarization into the M2 state and enhanced the proliferation of precancerous cell [42]. Fabregat et al. indicated that downregulated physiological proapoptotic molecules and overactivated antiapoptotic signals were important initiating factors for HCC [43]. In HCC, cell cycle progression is largely involved in the distortion disorder of cyclins or their regulators [44]. Some studies [45, 46] have found that the MAPK signaling pathway plays a central role in several steps of cancer development, including the development of cancer cell migration and apoptosis resistance. The involvement of T cell receptor (TCR) and costimulatory molecules is an important switch for T cell activation and plays an important role in tumor immunity [47, 48]. A study showed that baicalein and baicalin can stimulate T cell-mediated tumor immune response by reducing the expression of PD-L1 in HCC cells [49]. The results of GSEA enrichment analysis showed the cell cycle process, apoptosis pathway, the JAK-STAT signal pathway upstream of the MAPK signal pathway, and the NF-*κ*B pathway responding to TNF signal have some active differences between the obtained HCC samples and the normal samples.

Consequently, this study suggests that the therapeutic effect of SB on HCC is mainly achieved by targeting the p53 signaling pathway to regulate cell cycle progression, enhance tumor cell apoptosis, and regulate the MAPK signaling pathway and T cell-mediated tumor immunity, which are closely related to the occurrence and deterioration of HCC. It is worth noting that the results show that immune cells such as T cells play a certain role in SB treating HCC. However, the current research on HCC mainly focused on the relationship between SB and cell proliferation and apoptosis [33]. In the study of other diseases, it has been found that SB can regulate the immune function of macrophages [50–56], T cells [57–59], dendritic cells [58, 60], and mast cells [61–63], which suggests that the tumor immune mechanism of SB on HCC remains to be explored.

TCM treatment of diseases often have the overall network. Therefore, we constructed the PPI network for the obtained MEbrown module and screened out the core target CGRSB in the SB regulation and HCC network through the importance of the protein expressed by each gene in the network. These CGRSBs almost have the same positive expression pattern and upregulated in HCC tissues, which are adverse factors for patient survival. Among them, CDK2, AURKB, RRM2, CENPE, ESR1, and PRIM2 are of the highest importance. Studies have shown that [64] CDK2 is necessary for the proliferation of HCC cells and can promote the transformation of cells from G1 phase to S phase. AURKB, a member of the aurora kinase family, plays an important role in regulating cells from G2 to mitotic phase [65, 66]. The chromosome number of cells transfected with AURKB is unstable and characterized by invasive tumors [67]. RRM2 gene knockout can induce autophagy [68] in HCC cells. Human centromere-associated protein (CENPE) is one of the spindle checkpoint proteins, which has antitumor activity and can promote apoptosis of HCC cells [69]. The expression of PRIM2 is related to the survival rate of patients [70]. The above results show that these genes play an important role in the progress of HCC and can be used as potential biomarkers. The effect of the main components of SB on CGRSB has been confirmed in 40 studies, and most of them have the same regulatory effect on the target components. Among them, the role of PLCG1 and DDX55 has not been mentioned in the literature, which is worthy of further exploration (S5). Meanwhile, the docking results show that their binding is relatively stable, suggesting that the regulation of SB main components on HCC will involve core targets such as CDK2, and indirectly improved the survival of patients.

In order to further analyze the relationship between CGRSBs and tumor immunity in patients with HCC, by performing immune infiltration and correlation analysis, we found the proportion of immune cell infiltration such as T cells and macrophages was significantly correlated with the expression of CGRSBs, and these immune infiltration conditions had a certain impact on the survival of patients. In addition, single-cell analysis showed that CGRSBs was mostly concentrated in macrophages at the cellular level. Tumor-associated macrophages (TAM) are rich in HCC tissues, which is generally characterized by M2, namely, they inhibited immune function and promoted the pathogenesis of HCC [71]. The plasticity of TAM made the repolarization research from M2 to M1 become a hot topic in tumor immunotherapy [72]. Studies have shown that baicalin can reprogram into M1-like macrophages by initiating TAM and promote the production of proinflammatory cytokines, which is related to the increase of autophagy and the transcriptional activation of the RelB/p52 pathway [73]. Therefore, the mechanism of macrophages and T cells in the treatment of HCC by SB is worthy of further research.

In summary, cluster analysis of gene expression patterns at different time points was the first to be carried out in this study. The potential therapeutic targets for HCC were obtained. HCC-related MEbrown gene modules were obtained by WGCNA analysis. CDK2, AURKB, RRM2, CENPE, ESR1, and PRIM2 in its PPI network are the hub genes regulated by SB, which can be used as potential therapeutic targets for HCC. The model constructed by MEbrown module gene screening has a good predictive level in evaluating the prognosis of patients, which can provide suggestions for the prognosis of patients and facilitate the design of personalized treatment plans for patients. In addition, the results show that SB can not only regulate cell proliferation and apoptosis but also affect the infiltration of immune cells such as macrophages and T cells in tumor microenvironment to achieve the therapeutic effect by causing some certain effects, including the p53 signaling pathway, MAPK signaling pathway, apoptosis pathway, and T cell receptor-mediated tumor immunity, which provides a theoretical basis and direction for subsequent experimental research.

## Data Availability

The data used to support the results are available at TCGA (https://portal.gdc.cancer.gov/) and GEO (https://www.ncbi.nlm.nih.gov/geo/). The results of independent external validation and the files generated in the analysis process are available in Supplementary Materials.

## Supplementary Materials

Supplementary Materials Supplementary Table S1: single-cell analysis. Supplementary Table S2: GEO external dataset validation results. Supplementary Table S3: gene coefficient involved in model construction. Supplementary Table S4: cluster results of gene expression patterns under *Scutellaria baicalensis* treatment. Supplementary Table S5: literature mining on the interaction between CGRSB and SB main components .

## References

[1] Wong M. C., Jiang J. Y., Goggins W. B. (2017). International incidence and mortality trends of liver cancer: a global profile. *Scientific Reports*.

[2] Sung H., Ferlay J., Siegel R. L. (2021). Global cancer statistics 2020: GLOBOCAN estimates of incidence and mortality worldwide for 36 cancers in 185 countries. *CA: A Cancer Journal for Clinicians*.

[3] Fu J., Wang H. (2018). Precision diagnosis and treatment of liver cancer in China. *Cancer Letters*.

[4] Feng M., Pan Y., Kong R., Shu S. (2020). Therapy of primary liver cancer. *Innovation*.

[5] Ye L., Jia Y., Ji K. (2015). Traditional Chinese medicine in the prevention and treatment of cancer and cancer metastasis. *Oncology Letters*.

[6] Liao X., Bu Y., Jia Q. (2020). Traditional Chinese medicine as supportive care for the management of liver cancer: past, present, and future. *Genes & Diseases*.

[7] Ling C.-Q., Fan J., Lin H.-S. (2018). Clinical practice guidelines for the treatment of primary liver cancer with integrative traditional Chinese and Western medicine. *Journal of Integrative Medicine*.

[8] Jia W., Wang L. (2020). Using traditional Chinese medicine to treat hepatocellular carcinoma by targeting tumor immunity. *Evidence-based Complementary and Alternative Medicine*.

[9] Wang X., Wang N., Cheung F., Lao L., Li C., Feng Y. (2015). Chinese medicines for prevention and treatment of human hepatocellular carcinoma: current progress on pharmacological actions and mechanisms. *Journal of Integrative Medicine*.

[10] Zhao Q., Chen X.-Y., Martin C. (2016). Scutellaria baicalensis , the golden herb from the garden of Chinese medicinal plants. *Science Bulletin*.

[11] Cheng C.-S., Chen J., Tan H.-Y., Wang N., Chen Z., Feng Y. (2018). Scutellaria baicalensis and cancer treatment: recent progress and perspectives in biomedical and clinical studies. *The American Journal of Chinese Medicine*.

[12] Guotai W., Xingwu Y., Wang Q., Zhen W. (2016). Therapeutic effect of baicalin capsule combined with transcatheter arterial chemoembolization on primary hepatocellular carcinoma. *Chinese Journal of Integrated Traditional and Western Medicine on Liver Diseases*.

[13] Tomczak K., Czerwińska P., Wiznerowicz M. (2015). The Cancer Genome Atlas (TCGA): an immeasurable source of knowledge. *Contemporary Oncology*.

[14] Barrett T., Wilhite S. E., Ledoux P. (2012). NCBI GEO: archive for functional genomics data sets-update. *Nucleic Acids Research*.

[15] Ritchie M. E., Phipson B., Wu D. (2015). Limma powers differential expression analyses for RNA-sequencing and microarray studies. *Nucleic Acids Research*.

[16] Zhang F., Wu Y., Tian W. (2019). A novel approach to remove the batch effect of single-cell data. *Cell Discovery*.

[17] Kumar L., E Futschik M. (2007). Mfuzz: a software package for soft clustering of microarray data. *Bioinformation*.

[18] Langfelder P., Horvath S. (2008). WGCNA: an R package for weighted correlation network analysis. *BMC Bioinformatics*.

[19] Consortium G. O. (2004). The Gene Ontology (GO) database and informatics resource. *Nucleic Acids Research*.

[20] Kanehisa M., Furumichi M., Tanabe M., Sato Y., Morishima K. (2017). KEGG: new perspectives on genomes, pathways, diseases and drugs. *Nucleic Acids Research*.

[21] Therneau T. M., Lumley T. (2014). Package “survival”. *Survival analysis Published on CRAN*.

[22] Wu T. T., Chen Y. F., Hastie T., Sobel E., Lange K. (2009). Genome-wide association analysis by lasso penalized logistic regression. *Bioinformatics*.

[23] Bland J. M., Altman D. G. (1998). Statistics notes: survival probabilities (the kaplan-meier method). *BMJ*.

[24] Kleinbaum D. G., Klein M. (2012). Kaplan-Meier survival curves and the log-rank test. *Statistics for Biology and Health*.

[25] Fawcett T. (2006). An introduction to ROC analysis. *Pattern Recognition Letters*.

[26] Mering C. V., Huynen M., Jaeggi D., Schmidt S., Bork P., Snel B. (2003). STRING: a database of predicted functional associations between proteins. *Nucleic Acids Research*.

[27] Tang Y., Li M., Wang J., Pan Y., Wu F.-X. (2015). CytoNCA: a cytoscape plugin for centrality analysis and evaluation of protein interaction networks. *Biosystems*.

[28] Jumper J., Evans R., Pritzel A. (2021). Highly accurate protein structure prediction with AlphaFold. *Nature*.

[29] Kim S., Thiessen P. A., Bolton E. E. (2016). PubChem substance and compound databases. *Nucleic Acids Research*.

[30] Trott O., Olson A. J. (2010). AutoDock Vina: improving the speed and accuracy of docking with a new scoring function, efficient optimization, and multithreading. *Journal of Computational Chemistry*.

[31] Chen B., Khodadoust M. S., Liu C. L., Newman A. M., Alizadeh A. A. (2018). Profiling tumor infiltrating immune cells with CIBERSORT. *Methods in Molecular Biology*.

[32] Hankey B. F., Ries L. A., Edwards B. K. (1999). The surveillance, epidemiology, and end results program: a national resource. *Cancer Epidemiology and Prevention Biomarkers*.

[33] Bie B., Sun J., Guo Y. (2017). Baicalein: a review of its anti-cancer effects and mechanisms in Hepatocellular Carcinoma. *Biomedicine & Pharmacotherapy*.

[34] Xu M., Lu N., Zhang H. (2013). Wogonin induced cytotoxicity in human hepatocellular carcinoma cells by activation of unfolded protein response and inactivation of AKT. *Hepatology Research*.

[35] Hussain I., Waheed S., Ahmad K. A., Pirog J. E., Syed V. (2018). Scutellaria baicalensis targets the hypoxia‐inducible factor‐1*α* and enhances cisplatin efficacy in ovarian cancer. *Journal of Cellular Biochemistry*.

[36] Yu Y., Pei M., Li L. (2015). Baicalin induces apoptosis in hepatic cancer cells in vitro and suppresses tumor growth in vivo. *International Journal of Clinical and Experimental Medicine*.

[37] Ye F., Che Y., McMillen E. (2009). The effect ofScutellaria baicalensison the signaling network in hepatocellular carcinoma cells. *Nutrition and Cancer*.

[38] Wu X., Zhang H., Salmani J. M., Fu R., Chen B. (2016). Advances of wogonin, an extract from Scutellaria baicalensis, for the treatment of multiple tumors. *OncoTargets and Therapy*.

[39] Amaral J., Xavier J., Steer C., Rodrigues C. (2010). Targeting the p53 pathway of apoptosis. *Current Pharmaceutical Design*.

[40] Staib F., Perwez Hussain S., Hofseth L. J., Wang X. W., Harris C. C. (2003). TP53 and liver carcinogenesis. *Human Mutation*.

[41] Blagih J., Buck M. D., Vousden K. H. (2020). p53, cancer and the immune response. *Journal of Cell Science*.

[42] Lujambio A., Akkari L., Simon J. (2013). Non-cell-autonomous tumor suppression by p53. *Cell*.

[43] Fabregat I., Roncero C., Fernández M. (2007). Survival and apoptosis: a dysregulated balance in liver cancer. *Liver International*.

[44] Bisteau X., Caldez M., Kaldis P. (2014). The complex relationship between liver cancer and the cell cycle: a story of multiple regulations. *Cancers*.

[45] Kim E. K., Choi E. (2010). Pathological roles of MAPK signaling pathways in human diseases. *Biochimica et Biophysica Acta - Molecular Basis of Disease*.

[46] Zhou G., Yang J., Song P. (2019). Correlation of ERK/MAPK signaling pathway with proliferation and apoptosis of colon cancer cells. *Oncology Letters*.

[47] Thommen D. S., Schumacher T. N. (2018). T cell dysfunction in cancer. *Cancer Cell*.

[48] Hwang J.-R., Byeon Y., Kim D., Park S.-G. (2020). Recent insights of T cell receptor-mediated signaling pathways for T cell activation and development. *Experimental & Molecular Medicine*.

[49] Ke M., Zhang Z., Xu B. (2019). Baicalein and baicalin promote antitumor immunity by suppressing PD-L1 expression in hepatocellular carcinoma cells. *International Immunopharmacology*.

[50] Chen C.-Y., Shyue S.-K., Ching L.-C. (2011). Wogonin promotes cholesterol efflux by increasing protein phosphatase 2B-dependent dephosphorylation at ATP-binding cassette transporter-A1 in macrophages. *The Journal of Nutritional Biochemistry*.

[51] Xia K. (2006). Effect of total flavonoids in scutellaria Baicalensis georgi on mice peritoneal macrophage respiratory burst and COX-2 synthesis. *West China Medical Journal*.

[52] Kim O. S., Seo C.-S., Kim Y., Shin H.-K., Ha H. (2015). Extracts of Scutellariae Radix inhibit low-density lipoprotein oxidation and the lipopolysaccharide-induced macrophage inflammatory response. *Molecular Medicine Reports*.

[53] Wang J.-Y., Chuang H.-N., Chiu J.-H. (2006). Effects of Scutellaria baicalensis Georgi on macrophage-hepatocyte interaction through cytokines related to growth control of murine hepatocytes. *Experimental Biology and Medicine*.

[54] Chuang H., Wang J., Chiu J. (2005). Enhancing effects of Scutellaria baicalensis and some of its constituents on TGF-*β*1 gene expression in RAW 264.7 murine macrophage cell line. *Planta Medica*.

[55] Ali A., Kim E. H., Lee J.-H., Leem K.-H., Seong S., Kim W. (2021). Processed scutellaria baicalensis georgi extract alleviates LPS-induced inflammatory and oxidative stress through a crosstalk between NF-*κ*B and KEAP1/NRF2 signaling in macrophage cells. *Applied Sciences*.

[56] Yoon S.-B., Lee Y.-J., Park S. K. (2009). Anti-inflammatory effects of Scutellaria baicalensis water extract on LPS-activated RAW 264.7 macrophages. *Journal of Ethnopharmacology*.

[57] Jung S., Lee S.-Y., Choi D. (2017). Skullcap (Scutellaria baicalensis) hexane fraction inhibits the permeation of ovalbumin and regulates Th1/2 immune responses. *Nutrients*.

[58] Kim M. E., Kim H. K., Park H.-Y., Kim D. H., Chung H. Y., Lee J. S. (2013). Baicalin from Scutellaria baicalensis impairs Th1 polarization through inhibition of dendritic cell maturation. *Journal of Pharmacological Sciences*.

[59] Yang J., Yang X., Li M. (2012). Baicalin, a natural compound, promotes regulatory T cell differentiation. *BMC Complementary and Alternative Medicine*.

[60] Zhang H., Jiao Q., Gong Q., Zhang Y., Zhang W., Hu Z. Baicalin induced dendritic cell apoptosis in vitro. *Frontiers in Pharmacology*.

[61] Hsieh C., Hall K., Ha T., Li C., Krishnaswamy G., Chi D. S. (2007). Baicalein inhibits IL-1*β*-and TNF-*α*-induced inflammatory cytokine production from human mast cells via regulation of the NF-*κ*B pathway. *Clinical and Molecular Allergy*.

[62] Kubo M., Matsuda H., Kimura Y., Okuda H., Arichi S. (1984). Scutellariae Radix. X. Inhibitory effects of various flavonoids on histamine release from rat peritoneal Mast cells in vitro. *Chemical & Pharmaceutical Bulletin*.

[63] Choi Y. H., Han E. H., Chai O. H., Kim Y. K., Kim H. T., Song C. H. (2010). Scutellaria baicalensis inhibits mast cell-mediated anaphylactic reactions. *Korean Journal of Physical Anthropology*.

[64] Han X., Wang Z., Wang W., Bai R., Zhao P., Shang J. (2014). Screening on human hepatoma cell line HepG-2 nucleus and cytoplasm protein after CDK2 silencing by RNAi. *Cytotechnology*.

[65] Addepalli M. K., Ray K. B., Kumar B., Ramnath R. L., Chile S., Rao H. (2010). RNAi-mediated knockdown of AURKB and EGFR shows enhanced therapeutic efficacy in prostate tumor regression. *Gene Therapy*.

[66] Borah N. A., Reddy M. M. (2021). Aurora kinase B inhibition: a potential therapeutic strategy for cancer. *Molecules*.

[67] Tatsuka M., Katayama H., Ota T. (1998). Multinuclearity and increased ploidy caused by overexpression of the aurora- and Ipl1-like midbody-associated protein mitotic kinase in human cancer cells. *Cancer Research*.

[68] Yang P. M., Lin L. S., Liu T. P. (2020). Sorafenib inhibits ribonucleotide reductase regulatory subunit M2 (RRM2) in hepatocellular carcinoma cells. *Biomolecules*.

[69] He P., Hu P., Yang C., He X., Shao M., Lin Y. (2020). Reduced expression of CENP-E contributes to the development of hepatocellular carcinoma and is associated with adverse clinical features. *Biomedicine & Pharmacotherapy*.

[70] Mu R., Liu H., Luo S. (2021). Genetic variants of CHEK1, PRIM2 and CDK6 in the mitotic phase‐related pathway are associated with non‐small cell lung cancer survival. *International Journal Of Cancer*.

[71] Huang Y., Ge W., Zhou J., Gao B., Qian X., Wang W. (2021). The role of tumor associated macrophages in hepatocellular carcinoma. *Journal of Cancer*.

[72] Xiao H., Guo Y., Li B. (2020). M2-Like tumor-associated macrophage-targeted codelivery of STAT6 inhibitor and IKK*β* siRNA induces M2-to-M1 repolarization for cancer immunotherapy with low immune side effects. *ACS Central Science*.

[73] Tan H.-Y., Wang N., Man K., Tsao S.-W., Che C.-M., Feng Y. (2015). Autophagy-induced RelB/p52 activation mediates tumour-associated macrophage repolarisation and suppression of hepatocellular carcinoma by natural compound baicalin. *Cell Death & Disease*.

