# Supplementary material for: Targets and Potential Mechanism of Scutellaria baicalensis in Treatment of Primary Hepatocellular Carcinoma Based on Bioinformatics Analysis
Source: J Oncol. 2022 Feb 12;2022:8762717. doi: 10.1155/2022/8762717 (PMC8858046; doi:10.1155/2022/8762717)
Supplement: Supplementary Materials — Supplementary Table S1: single-cell analysis. Supplementary Table S2: GEO external dataset validation results. Supplementary Table S3: gene coefficient involved in model construction. Supplementary Table S4: cluster results of gene expression patterns under Scutellaria baicalensis treatment. Supplementary Table S5: literature mining on the interaction between CGRSB and SB main components . [file 8762717.f1.zip › 8762717.f1/Supplementary Table S1.pdf]

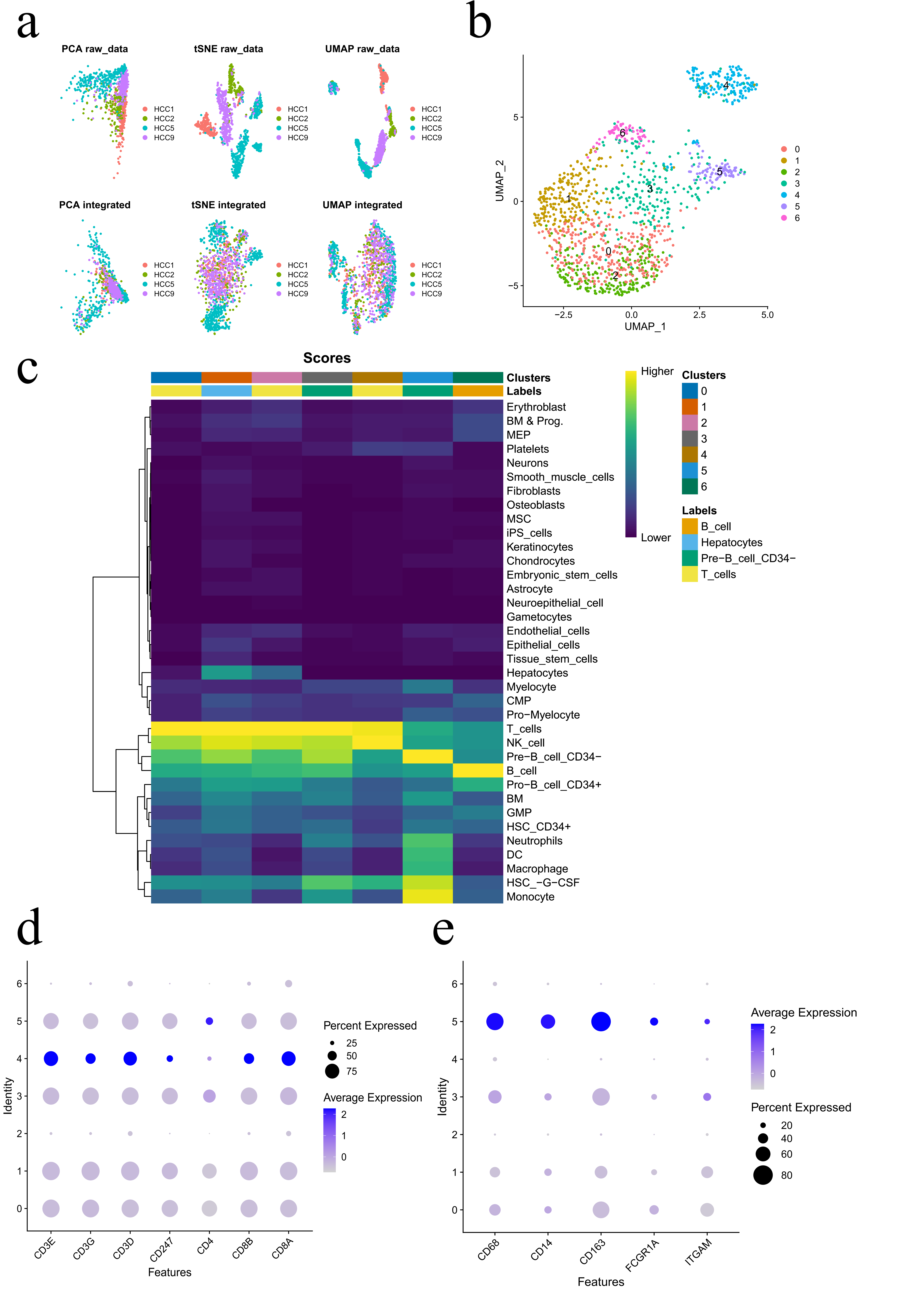

Supplementary Table S1: (a) PCA, TSNE, UMAP dimensionality reduction visualization after CCA integration. (b) Cluster distribution after dimensionlity reduction. (c) SingleR software annotation results. (d) Distribution of manually annotated T cell marker in cluster. (e) Distribution of manually annotated macrophage marker in cluster.
