# Supplementary material for: Targets and Potential Mechanism of Scutellaria baicalensis in Treatment of Primary Hepatocellular Carcinoma Based on Bioinformatics Analysis
Source: J Oncol. 2022 Feb 12;2022:8762717. doi: 10.1155/2022/8762717 (PMC8858046; doi:10.1155/2022/8762717)
Supplement: Supplementary Materials — Supplementary Table S1: single-cell analysis. Supplementary Table S2: GEO external dataset validation results. Supplementary Table S3: gene coefficient involved in model construction. Supplementary Table S4: cluster results of gene expression patterns under Scutellaria baicalensis treatment. Supplementary Table S5: literature mining on the interaction between CGRSB and SB main components . [file 8762717.f1.zip › 8762717.f1/Supplementary Table S2.docx]

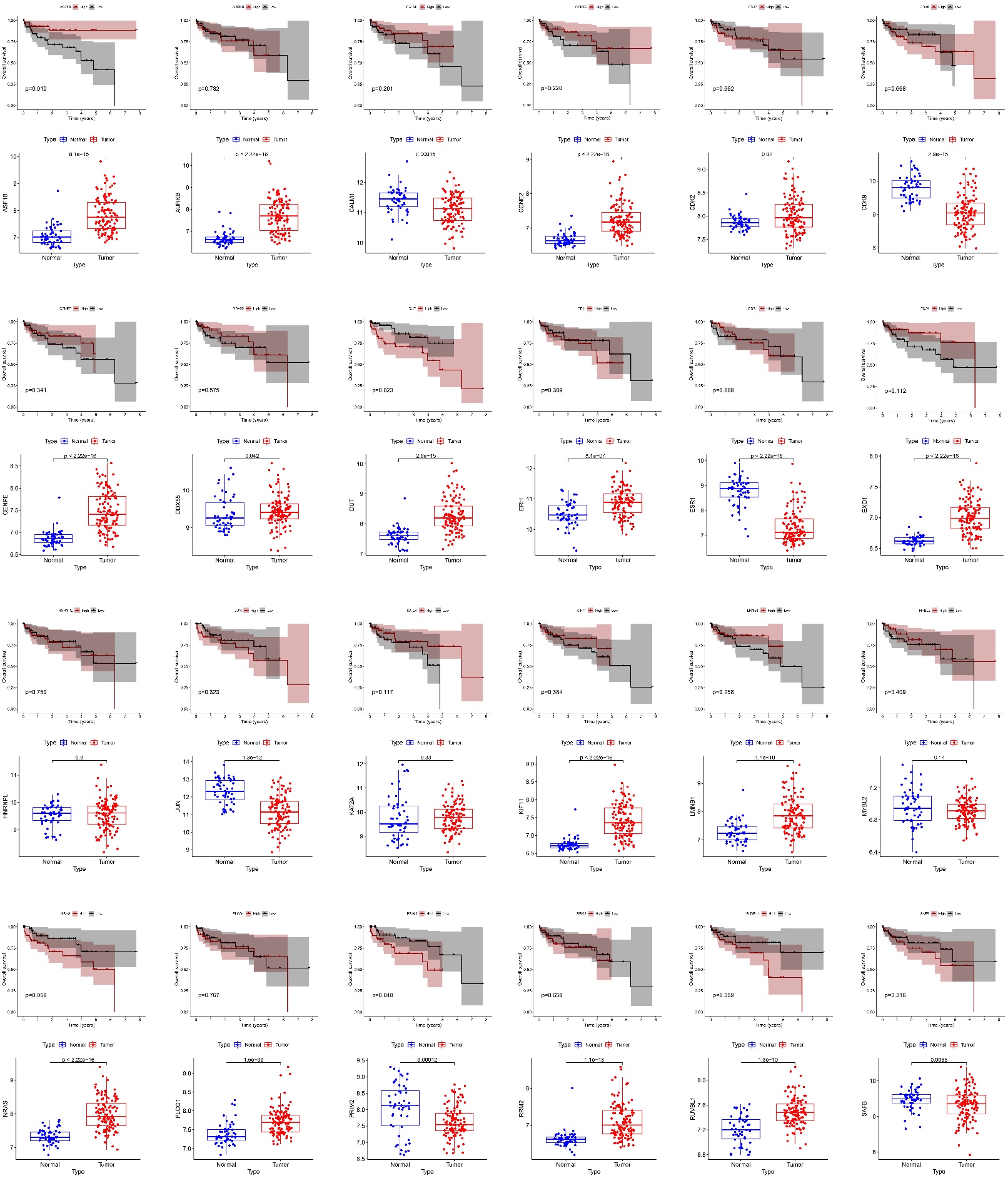
**GEO external set test results：**

Figure 1: Survival curve and differential box diagram of each gene in CGRSBs


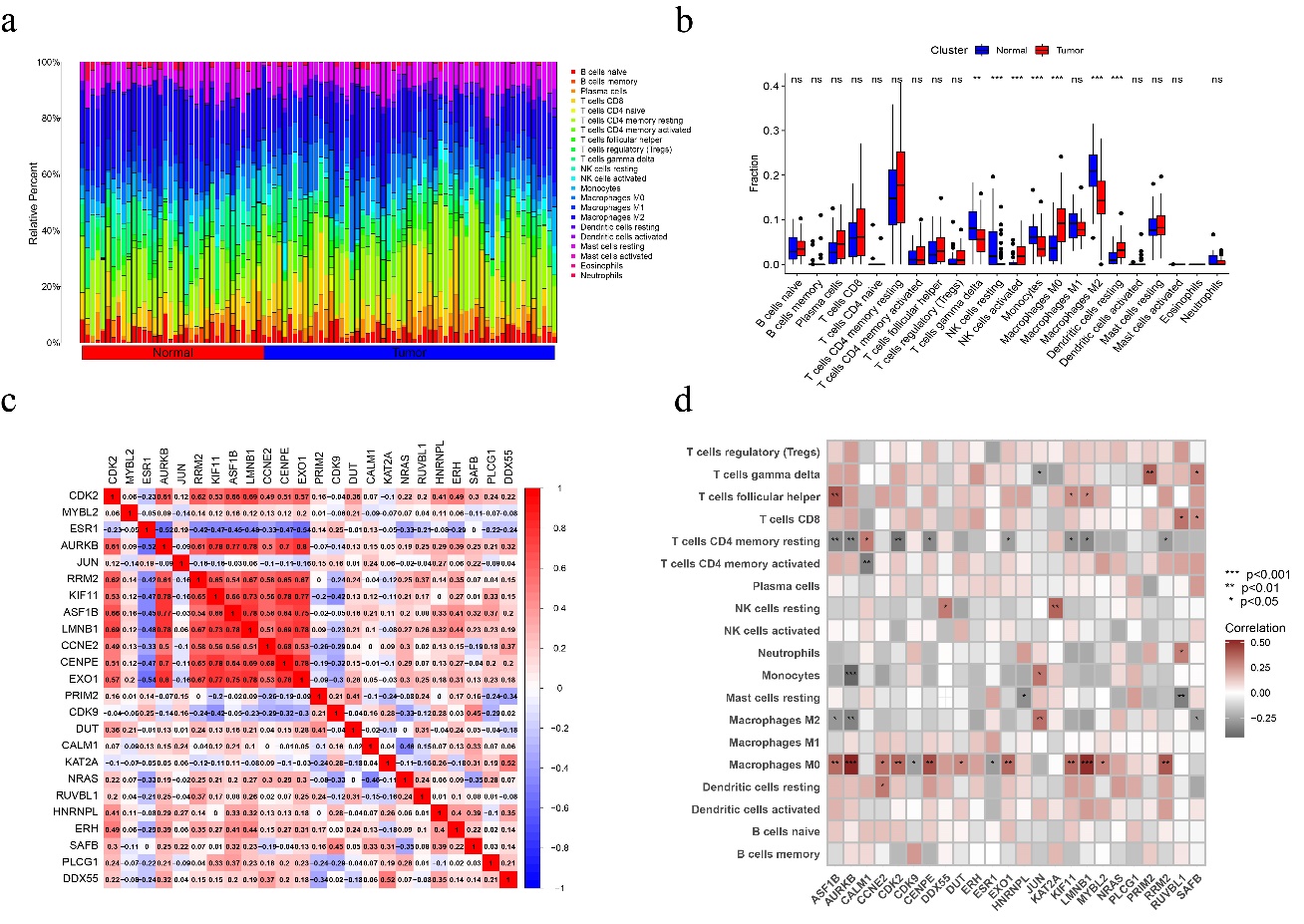


Figure 2: (a) Infiltration of different immune cells in HCC group and normal group. (b) Analysis of the difference of immune cell activity between HCC group and normal group. (c) Correlation diagram of co-expression of core genes, blue represents negative correlation, red represents positive correlation. (d) Heatmap of correlation between core genes and immune cells.


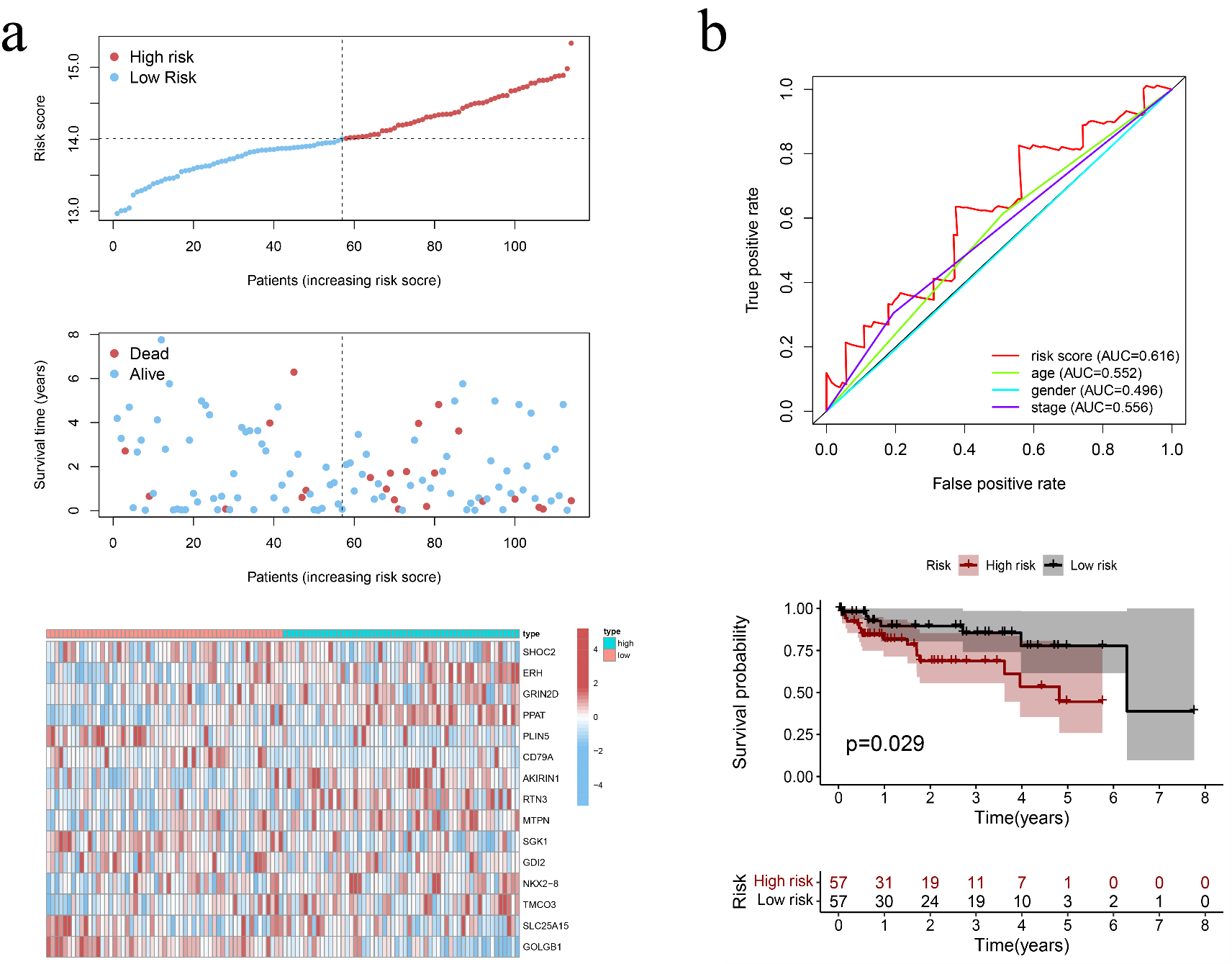
Figure 4: (a) Risk curve, survival and gene expression of different risk patients in GEO external set. (b) ROC map and survival curve of GEO external set.
