# Supplementary material for: Targets and Potential Mechanism of Scutellaria baicalensis in Treatment of Primary Hepatocellular Carcinoma Based on Bioinformatics Analysis
Source: J Oncol. 2022 Feb 12;2022:8762717. doi: 10.1155/2022/8762717 (PMC8858046; doi:10.1155/2022/8762717)
Supplement: Supplementary Materials — Supplementary Table S1: single-cell analysis. Supplementary Table S2: GEO external dataset validation results. Supplementary Table S3: gene coefficient involved in model construction. Supplementary Table S4: cluster results of gene expression patterns under Scutellaria baicalensis treatment. Supplementary Table S5: literature mining on the interaction between CGRSB and SB main components . [file 8762717.f1.zip › 8762717.f1/Supplementary Table S5.pdf]

Literature Mining on the Interaction between CGRSB and SB Main Components

ID	CDK2	MYBL2	ESR1	AURKB	JUN	RRM2	KIF11	ASF1B	LMNB1	CCNE2	CENPE	EXO1	PRIM2	CDK9	DUT	CALM1	KAT2A	NRAS	RUVBL1	HNRNPL	ERH	SAFB	PLCG1	DDX55
Baicalin	1-**	2+*	3-*	4-**	40-*	5-*	4-**	6-*	7-*	8-*	9-*	39-*	NA	4-**	NA	10-*	11+*	12-*	NA	13-*	NA	14-*	NA	NA
wogonoside	15-*	16-*	17-*	NA	18-*	19-*	NA	NA	NA	20+*	NA	NA	37-*	21-*	NA	NA	NA	22-*	NA	NA	NA	NA	NA	NA
Baicalein	23-**	11-*	3-*	4-**	24-*	19-*	25-*	26-*	2-*	8+*	27-*	39-*	NA	28-*	29-*	NA	NA	30-*	31-*	38-*	32-*	NA	NA	NA
wogonin	15-*	NA	36-*	NA	33-*	34-*	NA	26-*	NA	20+*	27-*	NA	37-*	28-*	NA	NA	NA	35-*	NA	NA	NA	NA	NA	NA

The serial number is a citation representing the source of the evidence. "+" and "-" respectively mean that the molecules treated by the main component of SB are up-regulated or down-regulated, "*" means that the research is other cancers, and "* *" means that the research is liver cancer. "NA" means that no related research has been found.

References:

[1].Yu, Y., M. Pei and L. Li, Baicalin induces apoptosis in hepatic cancer cells in vitro and suppresses tumor growth in vivo. Int J Clin Exp Med, 2015. 8(6): p. 8958-67.

[2].Kubatka, P., et al., Flavonoids against non-physiologic inflammation attributed to cancer initiation, development, and progression-3PM pathways. EPMA J, 2021. 12(4): p. 559-587.

[3].Chen, W., et al., Baicalin Promotes Mammary Gland Development via Steroid-Like Activities. Front Cell Dev Biol, 2021. 9: p. 682469.

[4].Huang, C., et al., AURKB, CHEK1 and NEK2 as the Potential Target Proteins of Scutellaria barbata on Hepatocellular Carcinoma: An Integrated Bioinformatics Analysis. Int J Gen Med, 2021. 14: p. 3295-3312.

[5].Ma, Z., et al., Traditional Chinese medicine-combination therapies utilizing nanotechnology-based targeted delivery systems: a new strategy for antitumor treatment. Int J Nanomedicine, 2019. 14: p. 2029-2053.

[6].Han, G., et al., Knockdown of anti-silencing function 1B histone chaperone induces cell apoptosis via repressing PI3K/Akt pathway in prostate cancer. Int J Oncol, 2018. 53(5): p. 2056-2066.

[7].Cao, W., et al., Effects of epigallocatechin gallate on the stability, dissolution and toxicology of ZnO nanoparticles. Food Chem, 2022. 371: p. 131383.

[8].Dai, Y., L. Sun and W. Qiang, A New Strategy to Uncover the Anticancer Mechanism of Chinese Compound Formula by Integrating Systems Pharmacology and Bioinformatics. Evid Based Complement Alternat Med, 2018. 2018: p. 6707850.

[9].Xiong, T., et al., PCAT-1: A Novel Oncogenic Long Non-Coding RNA in Human Cancers. Int J Biol Sci, 2019. 15(4): p. 847-856.

[10].Niu, K., et al., Molecular Targets and Mechanisms of Scutellariae radix-Coptidis rhizoma Drug Pair for the Treatment of Ulcerative Colitis Based on Network Pharmacology and Molecular Docking. Evid Based Complement Alternat Med, 2021. 2021: p. 9929093.

[11].Kabakov, A.E. and A.O. Yakimova, Hypoxia-Induced Cancer Cell Responses Driving Radioresistance of Hypoxic Tumors: Approaches to Targeting and Radiosensitizing. Cancers (Basel), 2021. 13(5).

[12].Aranha, E., et al., 22beta-hydroxytingenone induces apoptosis and suppresses invasiveness of melanoma cells by inhibiting MMP-9 activity and MAPK signaling. J Ethnopharmacol, 2021. 267: p. 113605.

[13].Ming, H., et al., Long non-coding RNAs and cancer metastasis: Molecular basis and therapeutic implications. Biochim Biophys Acta Rev Cancer, 2021. 1875(2): p. 188519.

[14].Qian, H., et al., Construction and Validation of an Autophagy-Related Prognostic Model for Osteosarcoma Patients. J Oncol, 2021. 2021: p. 9943465.

[15].Wang, Y., R.F. Yin and J.S. Teng, Wogonoside induces cell cycle arrest and mitochondrial mediated apoptosis by modulation of Bcl-2 and Bax in osteosarcoma cancer cells. Int J Clin Exp Pathol, 2015. 8(1): p. 63-72.

[16].Yuan, Y., et al., The Scutellaria baicalensis R2R3-MYB transcription factors modulates flavonoid biosynthesis by regulating GA metabolism in transgenic tobacco plants. PLoS One, 2013. 8(10): p. e77275.

[17].Li, M.Y., et al., Effects of Huangqin Decoction on ulcerative colitis by targeting estrogen receptor alpha and ameliorating endothelial dysfunction based on system pharmacology. J Ethnopharmacol, 2021. 271: p. 113886.

[18].Huang, Y., et al., A Systems Pharmacology Approach Uncovers Wogonoside as an Angiogenesis Inhibitor of Triple-Negative Breast Cancer by Targeting Hedgehog Signaling. Cell Chem Biol, 2019. 26(8): p. 1143-1158.e6.

[19].Hatami, E., et al., Gambogic acid: A shining natural compound to nanomedicine for cancer therapeutics. Biochim Biophys Acta Rev Cancer, 2020. 1874(1): p. 188381.

[20].Wang, Y., et al., Wogonin Induces Apoptosis and Reverses Sunitinib Resistance of Renal Cell Carcinoma Cells via Inhibiting CDK4-RB Pathway. Front Pharmacol, 2020. 11: p. 1152.

[21].Butt, G., et al., Regulation of cell signaling pathways by Wogonin in different cancers: Mechanistic review. Cell Mol Biol (Noisy-le-grand), 2021. 67(2): p. 1-7.

[22].Huang, L., et al., Baicalein and Baicalin Promote Melanoma Apoptosis and Senescence via Metabolic Inhibition. Front Cell Dev Biol, 2020. 8: p. 836.

[23].Hsu, S.L., et al., Baicalein induces a dual growth arrest by modulating multiple cell cycle regulatory molecules. Eur J Pharmacol, 2001. 425(3): p. 165-71.

[24].Su, M.Q., et al., Baicalein induces the apoptosis of HCT116 human colon cancer cells via the upregulation of DEPP/Gadd45a and activation of MAPKs. Int J Oncol, 2018. 53(2): p. 750-760.

[25].Miller, A.D., Lipid-based nanoparticles in cancer diagnosis and therapy. J Drug Deliv, 2013. 2013: p. 165981.

[26].Shen, Y.L., et al., Targeting cyclin-dependent kinase 9 in cancer therapy. Acta Pharmacol Sin, 2021.

[27].Kumar, M.S. and K.M. Adki, Marine natural products for multi-targeted cancer treatment: A future insight. Biomed Pharmacother, 2018. 105: p. 233-245.

[28].Polier, G., et al., Wogonin and related natural flavones are inhibitors of CDK9 that induce apoptosis in cancer cells by transcriptional suppression of Mcl-1. Cell Death Dis, 2011. 2: p. e182.

[29].Choi, J.S., et al., Solid dispersion of dutasteride using the solvent evaporation method: Approaches to improve dissolution rate and oral bioavailability in rats. Mater Sci Eng C Mater Biol Appl, 2018. 90: p. 387-396.

[30].Dou, J., et al., Baicalein and baicalin inhibit colon cancer using two distinct fashions of apoptosis and senescence. Oncotarget, 2018. 9(28): p. 20089-20102.

[31].Son, H.J., et al., 2,6-Dimethoxy-1,4-benzoquinone Inhibits 3T3-L1 Adipocyte Differentiation via Regulation of AMPK and mTORC1. Planta Med, 2019. 85(3): p. 210-216.

[32].Ding, F., et al., A review of the mechanism of DDIT4 serve as a mitochondrial related protein in tumor regulation. Sci Prog, 2021. 104(1): p. 36850421997273.

[33].Chen, L.G., et al., Wogonin, a bioactive flavonoid in herbal tea, inhibits inflammatory cyclooxygenase-2 gene expression in human lung epithelial cancer cells. Mol Nutr Food Res, 2008. 52(11): p. 1349-57.

[34].Pal, A. and R. Kundu, Human Papillomavirus E6 and E7: The Cervical Cancer Hallmarks and Targets for Therapy. Front Microbiol, 2019. 10: p. 3116.

[35].Zhao, K., et al., Wogonin suppresses melanoma cell B16-F10 invasion and migration by inhibiting Ras-medicated pathways. PLoS One, 2014. 9(9): p. e106458.

[36].Zhang, X.W., et al., [Mechanism of Mahuang Lianqiao Chixiaodou Decoction in treating eczema by network pharmacology and molecular docking technology]. Zhongguo Zhong Yao Za Zhi, 2021. 46(4): p. 894-901.

[37].Kumar, R., et al., Fascinating Chemopreventive Story of Wogonin: A Chance to Hit on the Head in Cancer Treatment. Curr Pharm Des, 2021. 27(4): p. 467-478.

[38].Hu, Q., et al., Baicalin and the liver-gut system: Pharmacological bases explaining its therapeutic effects. Pharmacol Res, 2021. 165: p. 105444.

[39].Huang, Z., et al., Baicalin-loaded macrophage-derived exosomes ameliorate ischemic brain injury via the antioxidative pathway. Mater Sci Eng C Mater Biol Appl, 2021. 126: p. 112123.

[40].Wan, Q., et al., Baicalin inhibits TLR7/MYD88 signaling pathway activation to suppress lung inflammation in mice infected with influenza A virus. Biomed Rep, 2014. 2(3): p. 437-441.
